# Antimicrobial and immunoregulatory effects of *Lactobacillus delbrueckii* 45E against genitourinary pathogens

**DOI:** 10.1186/s12929-023-00913-7

**Published:** 2023-03-23

**Authors:** Ameda Abdullah Bnfaga, Kai Wei Lee, Leslie Thian Lung Than, Syafinaz Amin-Nordin

**Affiliations:** 1 Department of Medical Microbiology, Faculty of Medicine and Health Sciences, Universiti Putra Malaysia, 43400 Serdang, Selangor Malaysia; 2 Department of Para-Clinic, Faculty of Medicine, Aden University, Aden, Yemen; 3 Hospital Sultan Abdul Aziz Shah, Universiti Putra Malaysia, Persiaran MARDI-UPM, 43400 Serdang, Malaysia

**Keywords:** *Lactobacillus delbrueckii*, *Lactobacillus reuteri* RC-14, Group B *Streptococcus*, *Escherichia coli*, *Klebsiella* spp., *Candida parapsilosis*, Vaginitis, Proinflammatory cytokine IL-17

## Abstract

**Background:**

Lactobacilli are essential microbiota that maintain a healthy, balanced vaginal environment. Vaginitis is a common infection in women during their reproductive years. Many factors are associated with vaginitis; one of them is the imbalance of microbiota in the vaginal environment. This study aimed to evaluate the antimicrobial properties of *Lactobacillus delbrueckii* 45E (*Ld45E*) against several species of bacteria, namely, Group B *Streptococcus* (GBS), *Escherichia coli*, *Klebsiella* spp., and *Candida parapsilosis*, as well as to determine the concentration of interleukin-17 (IL-17) in the presence of *Ld45E*.

**Methods:**

The probiotic characteristics of *Ld45E* were evaluated by examining its morphology, pH tolerance, adhesive ability onto HeLa cells, hemolytic activity, antibiotic susceptibility, and autoaggregation ability. Then, the antimicrobial activity of *Ld45E* was determined using *Ld45E* culture, cell-free supernatant, and crude bacteriocin solution. Co-aggregation and competition ability assays against various pathogens were conducted. The immunoregulatory effects of *Ld45E* were analyzed by measuring the proinflammatory cytokine IL-17. A p-value less than 0.05 was considered statistical significance.

**Results:**

*Ld45E* is 3–5 mm in diameter and round with a flat-shaped colony. pH 4 and 4.5 were the most favorable range for *Ld45E* growth within 12 h of incubation. *Ld45E* showed a strong adhesion ability onto HeLa cells (86%) and negative hemolytic activities. *Ld45E* was also sensitive to ceftriaxone, cefuroxime, ciprofloxacin, and doxycycline. We found that it had a good autoaggregation ability of 80%. Regarding antagonistic properties, *Ld45E* culture showed strong antimicrobial activity against GBS, *E. coli*, and *Klebsiella* spp. but only a moderate effect on *C. parapsilosis*. Cell-free supernatant of *Ld45E* exerted the most potent inhibitory effects at 40 °C against all genital pathogens, whereas bacteriocin showed a robust inhibition at 37 °C and 40 °C. The highest co-aggregation affinity was observed with GBS (81%) and *E. coli* (40%). Competition ability against the adhesion of GBS (80%), *E. coli* (76%), *Klebsiella* (72%), and *C. parapsilosis* (58%) was found. *Ld45E* was able to reduce the induction of the proinflammatory protein IL-17.

**Conclusions:**

*Ld45E* possessed antimicrobial and immunoregulatory properties, with better cell-on-cell activity than supernatant activity. Thus, *Ld45E* is a potential probiotic candidate for adjunct therapy to address vaginal infections.

## Background

Vaginal lactobacilli (LB) live in a symbiotic relationship modulated by female hormones that induce vaginal epithelium cells to secrete glycogen. Glycogen is metabolized by LB to produce lactic acid and keep the vagina at a constant pH of ≤ 4.5 [1, 2]. Different LB species exert antagonistic effects on several microorganisms, including pathogens, anaerobic microflora, numerous Gram negative *Enterobacteriaceae* groups, and Gram positive cocci that are not similar to *Lactobacilli* spp., such as Group B *Streptococcus* (GBS) [3]. Previous studies have described *Lactobacillus delbrueckii* spp. as a safe strain for their γ-hemolysis activity and sensitivity to some antibiotics. They also possess antimicrobial properties by secreting organic substances [4, 5] that inhibit the growth of some Gram negative bacteria, such as *Escherichia coli* and *Gardnerella* biofilm [3, 6]. The abilities of *L. delbrueckii* spp. to maintain the pH and adhere onto cell lines are conducive to forming biofilms and fending off vaginal pathogens [7].

Modulating innate immune responses can boost health and monitor chronic inflammatory bowel diseases. Correspondingly, the application of multistrain probiotic therapy demonstrates a significant regulator for innate immune homeostasis. Li et al. (2019) explained that the administration of *L*. *crispatus* and *L. delbrueckii* spp. could suppress the release of proinflammatory cytokines from the epithelial cells [8]. Furthermore, monitoring the secretion of proteins may reduce the ratio of certain cytokines that are produced by T helper cells, thereby arbitrating the adaptive immunity’s epithelial expressions to elevate IgG and promote antibody-mediated protection. The protective role of T helper 17 (Th17) cells to innate immunity is characterized by the secretion of interleukin (IL)-17 and IL-17F. It helps the host defend against pathogen infection and maintain the mucosal barrier, as well as induce tissue inflammation and autoimmune diseases [9].

Inappropriate antibiotic use can lead to bacteria drug resistance, whereas insufficient doses may result in an imbalanced LB levels, leading to recurrent infections [10]. According to Razzak et al., the predisposing risk factors of vaginitis refer to an inverse relationship between the presence of LB and the atiological agents of vaginal infection, including *Staphylococcus aureus*, *E. coli*, GBS, and *Klebsiella* spp. [11]. Furthermore, vaginitis treatment must be selective to avoid vaginal-microbiota disturbance. For these reasons, evaluating new prophylactic strategies is an imperative. Asymptomatic infection and non-infectious vaginitis render unsuitable the application of antimicrobials as treatment because the main problem refers to microbiota imbalance. Accordingly, an adjunct therapy to address vaginitis is required. The present research aimed to evaluate the *LB delbrueckii* strain 45E (*Ld45E*) as a potential adjunct-therapy probiotic candidate for vaginal infections pathogens by assessing its antimicrobial effects against GBS, *E. coli*, *Klebsiella* spp., and *Candida parapsilosis*. Hemolytic activity and antibiotic sensitivity tests were conducted to study the safety aspect of the strain. The adhesion capacity of *Ld45E* onto HeLa cells and autoaggregation, co-aggregation, and competition against pathogens provided potential information on the microorganism colonization of the vaginal tract. This research also explored the ability of *Ld45E* to suppress the IL-17 induction that controlled the proinflammatory response.

## Materials and methods

### Materials

#### Bacteria isolates

Table 1 shows the bacteria isolates used in this study. Previously isolated *Lactobacillus delbrueckii* strain 45E *Ld45E* from the anogenital region of a healthy Malaysian woman was maintained on de Man, Rogosa, and Sharpe (MRS) agar [12]. Pathogenic isolates of Group B *Streptococcus*, *Escherichia coli*, *Klebsiella* spp., and *Candida* *parapsilosis* were obtained from Universiti Putra Malaysia’s medical microbiology department. These bacterial collections were previously isolated from clinical samples using vaginal swabs, and ATCC species were commercially provided.

**Table 1:** Test pathogens utilized in this study

Microorganism strains	Reference number
Group B* Streptococcus*	Clinical isolate
*Escherichia coli*	Clinical isolate
*Klebsiella* spp*.*	Clinical isolate
*Candida parapsilosis*	Clinical isolate
Group B* Streptococcus*	ATCC 8017
*Escherichia coli*	ATCC 25922
*Klebsiella* spp*.*	ATCC 3566
*Candida parapsilosis*	ATCC 3434
*Lactobacillus reuteri*	RC-14 (commercial control)

#### Bacterial-culture maintenance

Inocula of GBS, *E. coli*, *Klebsiella*, *C. parapsilosis*, *Ld45E*, and *L. reuteri* RC-14 (∼10^8^ colony-forming units (CFU)/mL) were cultured overnight for 24–48 h at 37 °C in tryptic soy broth, Luria broth, Sabouraud dextrose broth, and MRS broth (Himedia, India), respectively. The stocks were frozen at − 80 °C in a sterilized mixture of glycerol (v/v). Glycerol stocks’ were maintained every six months by subculturing the frozen stock.

#### Culture of HeLa cells

HeLa cells were slowly defrosted, added to 5 mL of Dulbecco’s minimal essential medium (DMEM; Sigma–Aldrich, USA) in a falcon tube, and centrifuged for 4 min at 1200 rpm. The pellet was transferred into a mixture of DMEM solution supplemented with 10% of fetal bovine serum (GenClone, USA) and 1% of penicillin G/streptomycin (10,000 IU/mL to 10,000 μg/mL) (ThermoFisher Scientific, USA). About 5 mL of the mixture was seeded into a T-flask 25 cm^2^ for 24 h at 37 °C in a 5% CO_2_ incubator. The old medium was aspirated, and the cells were washed three times with 2 mL of PBS to remove debris [13, 14]. About 1 mL of 0.25% trypsin–EDTA solution (Sigma–Aldrich, USA) was added to detach HeLa cells from the flask for cell calculation. About 1 × 10^5^ HeLa cells/mL were seeded onto a 24-well tissue-culture plate in a 5% CO_2_ incubator for 24 h at 37 °C until a monolayer of cells reached (~ 90% confluence) a density of 2 × 10^5^ cells/well [13, 14]. The growth medium was diskharged, and the cells were washed twice with PBS to be used in *Ld45E* adhesion assays.

#### Preparation of cell-free supernatant (CFS)

Overnight cultures at exponential-growth phase were incubated anaerobically in MRS broth for 24 h at 37 °C and *Ld45E* cells were harvested by centrifugation at 12,000 × *g* for 10 min at 4 °C. The supernatant was aseptically filtrated through a 22 µm millipore to obtain a sterile CFS. The CFS was dispensed into three vials and labeled as CFS1, CFS2, and CFS 3. Then, CFS 1 was incubated for 24 h at 37 °C, CFS 2 at 40 °C, and CSF 3 at 50 °C.

#### Preparation of crude bacteriocin (CB) solution (CBS)

Similarly, the previous filtrate of the CFS solution was centrifuged at 4000 rpm for 20 min at 4 °C. The supernatant was adjusted with NaOH to achieve a pH 7.0 concentration. Bovine serum (1 mL) was added to inhibit the active hydrogen peroxide [15]. The supernatant was filtered through 0.45 µm Millipore (Advantec Co., Japan). Precipitation was achieved by adding ammonium sulfate (1:1) concentration, and the resulting pellets were resuspended in phosphate-buffered saline (PBS) (pH 7.0) for CB1 solution [16]. Three heat-stable solutions were obtained at 37 °C, 40 °C, and 50 °C.

### Characterization of *Ld45E*

#### Morphology

Gram staining protocol was followed for the initial screening of *Ld45E* size and compared with the control group *L. reuteri* RC14. *Ld45E* was incubated overnight with different pathogen strains. A thin smear of the bacterial mixture was placed on a glass slide. A primary crystal violet stain was applied to a heat-fixed smear, followed by adding iodine with rapid decolorisation with alcohol. Safranin was finally added, and the slide was screened under a light microscope. The presence of peptidoglycan in the Gram positive bacterial cell wall retained the crystal violet stain and prevented iodine diffusion owing to the closure of pores in the cell wall. Gram positive bacteria appeared purple and Gram negative bacteria were red. The physical shape of *Ld45E* was compared with that of the control strain *L. reuteri* RC-14. The results were reported as large, thick (0.5–0.9 µm), and long bacilli (3–9 µm), or tiny, thin (0.2–0.6 µm), and short bacilli (2–5 µm) under optical light microscopy (100 × magnitude) [17, 18].

#### pH tolerance of *Ld45E*

The viability of *Ld45E* within different pH ranges were measured to determine the pH tolerance of *Ld45E*. Normal female vaginal pH and vaginal candidiasis pH were equivalent to 3.5–4.5, whereas bacterial vaginosis pH was > 4.5. [19]. An overnight culture of *Ld45E* in MRS broth at pH 6.6 was centrifuged at 5000 rpm for 15 min at 4 °C and rinsed twice with PBS. The solution was adjusted to 0.5 OD_600_ nm, approximately 5 × 10^7^ CFU/mL, to standardize the growth of LB cells [5, 20]. About 1 mL of *Ld45E* was subcultured into 15 mL of MRS broth. The pH was adjusted to 3.0–7.0 by using 1 M acetic acid and 1 M NaCl (Merck, Germany), analogous to the vaginal secretions indicated by Owen and Katz [21]. *Ld45E* was incubated anaerobically at 37 °C for 24 h at three time intervals to determine the estimated viability time in the vagina and the compared it with that of the control strain *L. reuteri* RC-14. *Ld45E* growth was monitored by spectrophotometric absorbance at OD_600_ nm and serial-dilution plating technique where the strain was incubated anaerobically at 37 °C for 48 h.

#### Adhesion capacity of *Ld45E* on HeLa cells

Adhesion assay was performed as described by Carmo et al., 2016 with some modifications [22]. An overnight *Ld45E* culture was centrifuged at 6000 × *g* for 15 min and washed two times with PBS. The pellet or precipitant was resuspended in 500 µL of DMEM (Sigma–Aldrich, USA). Approximately 100 µL of *Ld45E* suspension (5 × 10^7^ CFU/mL) were added onto 24-well plates and incubated for 3 h at 37 °C in a 5% CO_2_ incubator. Afterwards, each well of the 24-well plate was washed with PBS three times to eliminate the unattached *Ld45E*. The adhered quantity of *Ld45E* on HeLa cells was determined by adding 500 µL of 0.25% trypsin–EDTA solution (Sigma–Aldrich) to allow cell detachment. Wells with HeLa cells + *Ld45E* suspensions were then incubated for 10 min at 37 °C, and the suspension was used for appropriate serial dilutions. About 100 µL was plated out on MRS agar and incubated for 48 h at 37 °C in a 5% CO_2_ incubator [22]. *L. reuteri* RC-14 was used as a positive control, and HeLa cells alone were the negative control. Lastly, a Gram stain technique was used to visualize the adhesion of *Ld45E* on HeLa cells and viewed under an inverted microscope. Adherence percentage (%) was calculated by comparing the number of adherent bacterial cells onto the total cell number of the added bacterial suspension (5 × 10^7^ CFU/mL) expressed by the following formula:

$${\text{Adherence}}\;{\text{percentage}} \, (\%) = \frac{{{\text{Number}}\;{\text{of}}\;{\text{adherent}}\;{\text{bacterial}}\;{\text{cells}}\;({\text{CFU}}\;{\text{mL}}^{{{-}{1}}} )}}{{{\text{Total}}\;{\text{bacterial}}\;{\text{cell}}\;{\text{number}}}} \times 100\%$$

#### Hemolytic activity of *Ld45E*

Overnight *Ld45E* was grown in MRS broth at 37 °C and then streaked over a Columbia blood agar plate (CM0331, Oxoid). Incubation was under anaerobic conditions for 48 h at 37 °C. The hemolytic reaction was determined visually and distinguished as either a greenish discoloration around the colony (known as α-hemolysis or partial hemolysis), or a distinct lysed zone around the colony (known as β-hemolysis or complete hemolysis), or no changes could occur in the agar medium (known as γ-hemolysis or no hemolysis). In the hemolytic analysis, GBS (clinical isolates and ATCC 8017) were β-hemolytic strains used as the positive control group. *Enterococcus* was used as the negative control [23].

#### Antibiotic susceptibility of *Ld45E*

*Ld45E* was subcultured anaerobically on MRS broth for 24 h at 37 °C. The suspensions were adjusted to 0.5 OD_600_ nm equivalent to 1.5 × 10^8^ CFU/mL. Dissemination of 100 µL of *Ld45E* was overlaid on the surface of MRS agar plates. Four disks were placed on the agar surface per Petri dish and incubated for 24 h at 37 °C in an anaerobic jar. Antimicrobial susceptibility test was performed according to the disk-diffusion method described by Barry et al. (1970) for clinical isolates [24]. Antimicrobial sensitivity (in triplicates) was measured following a modified disk-diffusion method of the National Committee for Clinical Laboratory Standards [25]. About 20 mL of melted MRS agar was poured into a Petri dish (9 cm). Petri plates were left to solidify at room temperature for 15 min before dispensing LB and antibiotic disks. After anaerobic incubation for 24 h at 37 °C, the inhibition-zone diameter was measured. The outcomes were expressed in terms of inhibition-zone diameter (mm), resistance (R), intermediate (I), and susceptible (S) based on the interpretative standards described by CLSI [26, 27].

*L. reuteri* RC-14 (as an LB strain) and GBS (as a Gram positive strain) were selected to be the control strains for their susceptibility to the antibiotics of interest and ability to grow anaerobically on the agar.

#### Autoaggregation assay

Autoaggregation assay was performed according to Aslim et al. 2017 [28]. We centrifuged a 48 h culture of *Ld45E* in MRS broth at 10,000 × *g* for 15 min at 4 °C and washed twice in PBS (pH 7.5). The harvested LB pellet was adjusted to a density of 0.5 OD_600 nm_ and observed under a spectrophotometer (BioPhotometer plus, Eppendorf, USA). *Ld45E* was then resuspended in PBS and incubated for 4 h at room temperature. The control tube containing 2 mL of *L. reuteri* RC-14, at a final concentration of 0.5 OD_600_ nm, was incubated for 4 h at room temperature. The reproducibility of the obtained results was verified in three independent assays. The percentage of autoaggregation was measured by the formula:

$${\text{Auto-aggregation}} \, (\%) = \left[ {\left( {{\text{OD1}}{-}{\text{OD2}}} \right)/\left( {{\text{OD1}}} \right)} \right] \times 100$$

where OD1 is the first optical density (OD) of *Ld45E*, and OD2 is the OD of *Ld45E* after 4 h. The bacterial aggregation score was confirmed after 4 h of incubation under a light microscope. Good autoaggregation of *Ld45E* was expressed as (+ +) of precipitate forming with no turbidity in the solution, whereas no autoaggregation (−) indicated turbid solution and no precipitate forming. Mixed autoaggregation ( ±) showed both a precipitate and constant turbidity [28, 29].

### Antimicrobial activity of *Ld45E*

#### Antagonistic effect of *Ld45E* culture using agar plug-diffusion method

LB strains were cultured in 20 mL of MRS agar and left to grow anaerobically for 48 h at 37 °C before the experiment. Agar plug diffusion was performed according to Balouiri et al. method [30]. Suspensions of *Ld45E* were adjusted to reach 0.5 OD_600_ nm equivalents to 1.5 × 10^8^ CFU/mL to standardize the growth. Then, 0.1 mL of *Ld45E* was inoculated into 500 mL of melted MRS agar at 37 °C [31]. The mixture was poured into a sterile Petri dish. When the inoculated agar solidified, holes about 8 mm in diameter were cut on the agar with a sterile cork borer. Each plug containing *Ld45E* was deposited on the Mueller–Hinton (MH) agar surface of another plate that was previously spread with the pathogen isolates. Overnight cultured pathogens GBS*, E. coli*, *Klebsiella* spp*.,* and *C. parapsilosis* were standardized to 0.5 OD_600_ nm by using a spectrophotometer (BioPhotometer plus, Eppendorf, USA) before being cultured into fresh agar. An uncultured MRS agar plate was used as a (blank/negative control) to confirm no contamination with microbial growth, and *L. reuteri* RC-14 served as a positive control. About 1 mL of each culture was plated and incubated for 24 h at 37 °C. The assay was performed in triplicate for three independent assays per isolate. The diameters of inhibition zones were measured and recorded in the following days. The inhibition index was calculated by applying the following equation [30]

$${\text{IP = }}\frac{{\Phi \;{\text{Clear}}\;{\text{Zone}} - \Phi \;{\text{Plug}}\;{\text{Size}}}}{{\Phi \;{\text{Plug}}\;{\text{Size}}}}$$

where IP is the inhibition index, and Ф is diameter (mm).

#### Antagonistic effect of *Ld45E* CFS and bacteriocin using well-diffusion methods

Determination of the antimicrobial efficacy of *Ld45E* CFS and bacteriocin solution against genital pathogens was performed through a well-diffusion assay described by Balouiri et al. [30] and the modified protocol by Sharma et al. [32]. An aliquot of each tested pathogen was spread-plated over pre-solidified plates. The inoculated sterile borer was used to equidistant 8 mm wells. About 100 µL of CFS 1 was dispensed into each pre-labeled well and then incubated for 24 h at 37 °C to obtain the 1st heat-stable CFS solution. The second CFS2 vial was kept in an oven for 24 h at 40 °C to obtain the second heat-stable CFS solution. CFS3 was incubated for 24 h at 50 °C for the third heat-stable CFS solution before dispensing into a well-diffusion assay. About 100 μL of MRS broth was used as a negative control and a similar amount of *L. reuteri* RC-14 as a positive control.

### Coaggregation and competition assays

#### Co-aggregation assay

The assessment of the co-aggregation ability of the *Ld45E* strain with clinically isolated pathogens was determined as described by Aslim et al. [28]. Similarly, as in autoaggregation preparation, 1:1 mL of *Ld45E* suspension was mixed in a vortex for 10 s and incubated for 4 h at room temperature in pairs with the bacterial pathogens. The concentration of the suspension was equivalent to 0.5 OD_600_ nm at a spectrophotometer. The control tube contained 2 mL of the suspension mixture of *L. reuteri* RC-14: pathogen (1:1) mL, with a density of 0.5 at OD_600_ nm after 4 h of incubation at room temperature. The results were verified by carrying out triplicates of each suspension in three independent assays. The following formula was used to express the co-aggregation:

$${\text{Co-aggregation}}\;(\%) = \frac{{[({\text{A}}_{{{\text{Lac}}}} + {\text{ A}}_{{{\text{path}}}} )/{2}{-}{\text{A}}_{{{\text{mix}}}} ]}}{{\left( {{\text{A}}_{{{\text{LAB}}}} + {\text{A}}_{{{\text{path}}}} } \right)/{2}}}$$

where **OD**_**Path**_ is the OD of pathogens, represented by GBS*, E. coli, Klebsiella*, and *C. parapsilosis*; **OD**_**Lact**_ is the OD of the LB strains; **OD**_**mix**_ is the OD of the co-cultured mixture of LB strains and pathogen strains after 4 h of incubation.

#### Competition assay

A suspension of *Ld45E* and pathogens cultured overnight of approximately 1 mL (5 × 10^7^ CFU/mL) of *Ld45E* + pathogens was added to each well of a previously seeded 24-well plate with HeLa cells and incubated for 24 h at 37 °C in a 5% CO_2_ incubator. Non-adhered bacterial cells were washed out three times with PBS (pH 7.5). HeLa cells and the bacterial cells were detached by adding 500 µL of 0.25% trypsin–EDTA solution (Sigma–Aldrich) into (HeLa–LB–pathogen) wells, and the plate was incubated for 5 min at 37 °C. The numbers of viable *Ld45E* and the pathogenic that remained on HeLa cells were determined by plating the bacteria on MRS agar and selective media after serial dilutions. Serial dilution factors were varied according to the *Ld45E* concentration that competed with other pathogens on HeLa cells. The results were expressed according to the LB that adhered onto the inhibited rate of pathogenic bacteria per well by following the competition formulation [33]. Three independent assays were conducted to confirm the reproducibility of the results.

$${\text{Inhibition}}\;{\text{rate}}\;{\text{of}}\;{\text{pathogen}}\;{\text{incubated}}\;{\text{with}}\;{\text{lactobacilli}} = \left[ {{1} - \left( {N_{1} /N_{0} } \right)} \right] \times {1}00$$

where *N*_1_ is the number of pathogens adhered onto HeLa cells, and *N*_0_ is the number of initial pathogens added into the wells of a 12-well plate.

### Effect of *Ld45E* on IL-17 secretion

LB *Ld45E* and the control strain *L. reuteri* RC-14 were incubated with HeLa cells for 24 h. The overnight solution served as a control, and the solution of overnight HeLa cells alone served as a blank. Similarly, the overnight solutions of pathogens incubated with *Ld45E* were transferred to a 96-well plate, respectively, for ELISA reading. A microplate reader read the absorbance under a spectrophotometer at OD_450_ nm. The density of the yellow-colored product was proportional to the target amount of sample captured in the plate. The standard curve was plotted as the relative absorbance OD_450_ nm of the target concentrations (X) vs. the standard solution (Y) of the value provided by the company protocol.

### Data analysis

SPSS statistical analysis package (version 21.0 for Windows) was used to analyze the significance of the results. An independent t-test was used to compare two means, and ANOVA was used to compare more than two means. A p-value less than 0.05 was considered statistical significance.

## Results

### Characteristics of *Ld45E* probiotic profile

#### Morphology

**Fig. 1:**
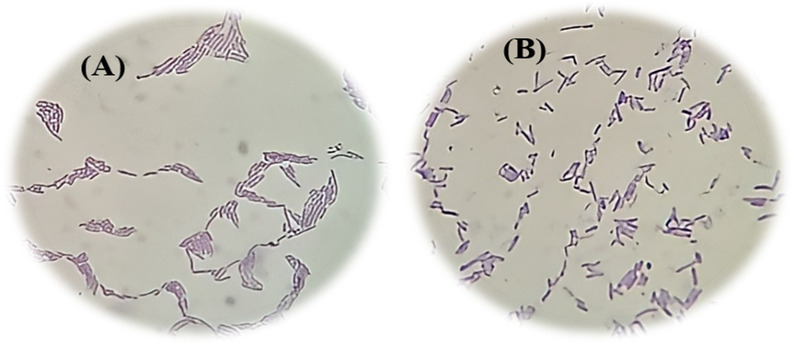
**A** Gram stain of *Ld45E* shows large long purple bacilli mostly in clusters under light microscope magnification 100 ×. **B** Gram stain of *L. reuteri* RC-14 shows tiny short purple bacilli in a single form under light microscopy (magnification 100 ×)

LB strains were subcultured on MRS agar at pH 6.5. *Ld45E* had relatively bigger creamy white colonies 3–5 mm in diameter, dry and rounded flat shape compared with the control *L. reuteri* RC-14. The control strain had smaller colonies of 2–4 mm in diameter with a shiny white appearance and smooth mucoid round shape. *Ld45E* was categorized under the large LB strains, and *L. reuteri* RC-14 was relatively small bacilli, as shown in Fig. 1. Interestingly, the cell size was the primer for *Ld45E* that promoted the strong aggregation properties of the strain.

#### pH tolerance of *Ld45E*

**Fig. 2:**
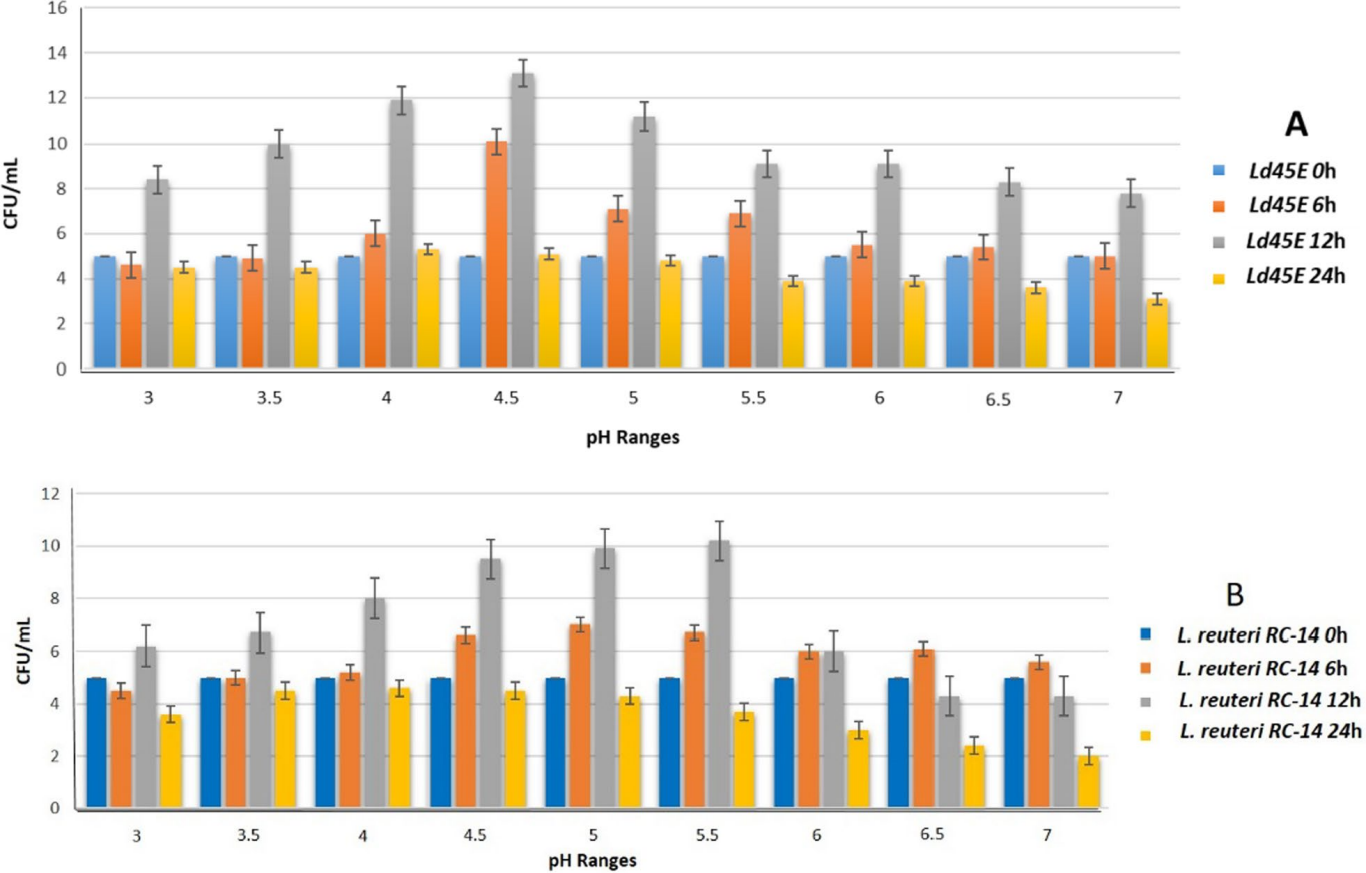
Stability of **A** *Ld45E* under pH conditions simulating those in the human vagina at three time intervals (6, 12, and 24 h), compared with **B** *L. reuteri* RC-14 (control)

The stability of LB at a pH medium allows them to live longer and stand different pH changes in the host body. The control of bacterial growth was adequate to 0.5 ± at OD_600_ nm, equivalent to 5 × 10^7^ CFU/mL. The growth of *Ld45E* decreased slightly than the initial solution (4.6 × 10^7^ and 4.9 × 10^7^) at low pH ranges (3 and 3.5), and the mean at the acidic pH (3 and 3.5) indicated the lowest proliferation rate at 6 h of incubation, whereas *Ld45E* showed good proliferation at pH ranges (4–7). Incubation of *Ld45E* for 12 h showed constant proliferation at all pH ranges, estimating that the strain adapted to the medium and was able to withstand the acidic pH (3 and 3.5) within 12 h. Prolonged incubation of *Ld45E* for 24 h indicated the tolerance for pH 4 and 4.5 only, and growth was slightly reduced at pH 3 (4.5 × 10^7^) and pH 3.5 (4.5 × 10^7^). *Ld45E* was unable to tolerate other pH ranges (5–7) within 24 h and showed a noticeable growth reduction, as shown in Fig. 2A. Thus, *Ld45E* could tolerate different pH ranges better than the control strain *L. reuteri* RC-14 (Fig. 2B), in accordance with the time rather than the concentration, and pH 4 and 4.5 were the favorable values to grow within 12 h of incubation.

### Adhesion ability of *Ld45E* onto HeLa cells

**Fig. 3:**
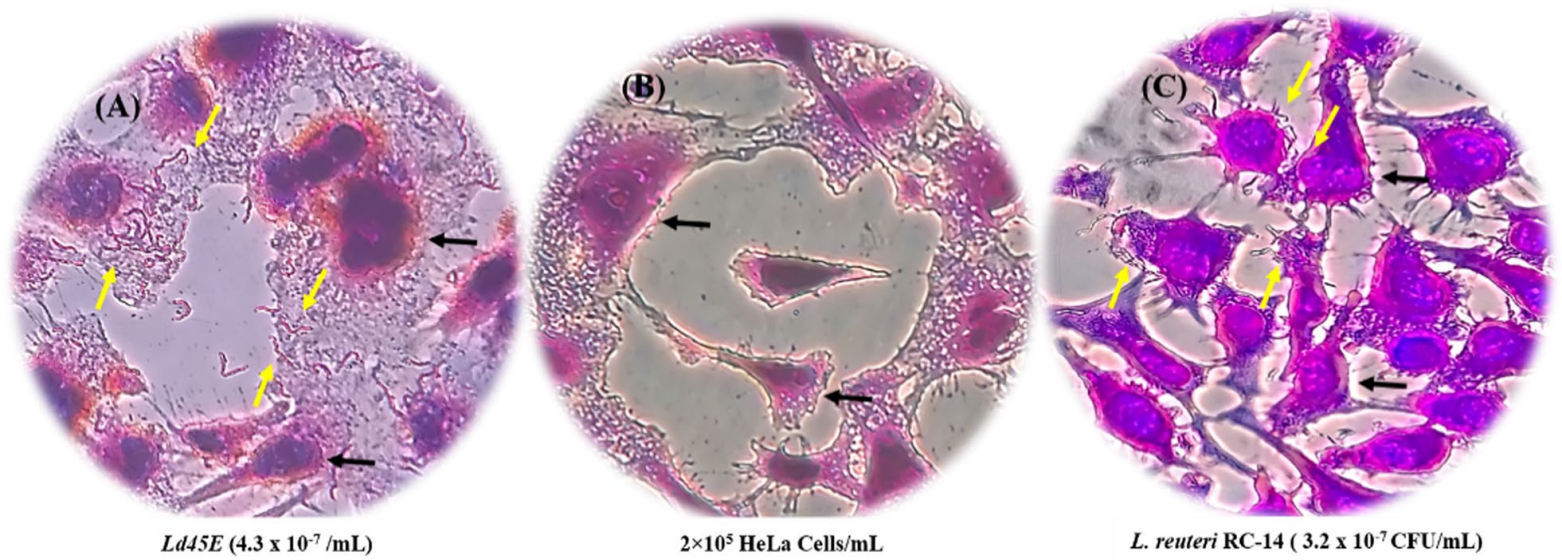
Inverted microscopy was used to visualize the adhesion of LB on HeLa cells. **A** shows *Ld45E* (purple bacilli) on HeLa cells. The yellow arrows indicate a high quantity of *Ld45E* adhered onto HeLa cells. **B** Shows the control group HeLa cells incubated for 24 h at 37 °C in a 5% CO_2_ incubator. The black arrows indicate (purple cells) stained with crystal violate. **C** shows *L. reuteri* RC-14 (purple bacilli) on HeLa cells. The yellow arrows indicate a few *L. reuteri* RC-14 adhered onto HeLa cells (purple stain), magnification 40 ×

LB adhesion is an intrinsic property that allows them to adhere onto the cell surface, reducing the multiplication and excluding the attachment of pathogens. The adhesion feature is essential in selecting probiotic candidates because it facilitates the proliferation of LB. It also maintains a balanced flora in the organs by preventing their instant elimination of the membrane peristalsis or wave-like contractions of the epithelial. In this research, HeLa cells were used to mimic the vaginal medium, and *Ld45E* demonstrated a strong adhesion onto HeLa cells 4.3 × 10^7^ CFU/mL. Almost 86% of *Ld45E* were capable of attaching to HeLa cells out of the original concentration of 5 × 10^7^ CFU /mL. The control group *L. reuteri* RC-14 showed a low adhesion capacity with a recovery of 3.2 × 10^7^ CFU/mL equivalent to (64%) within 3 h of the incubation period, as shown in (Fig. 3).

#### Hemolytic activity

**Fig. 4:**
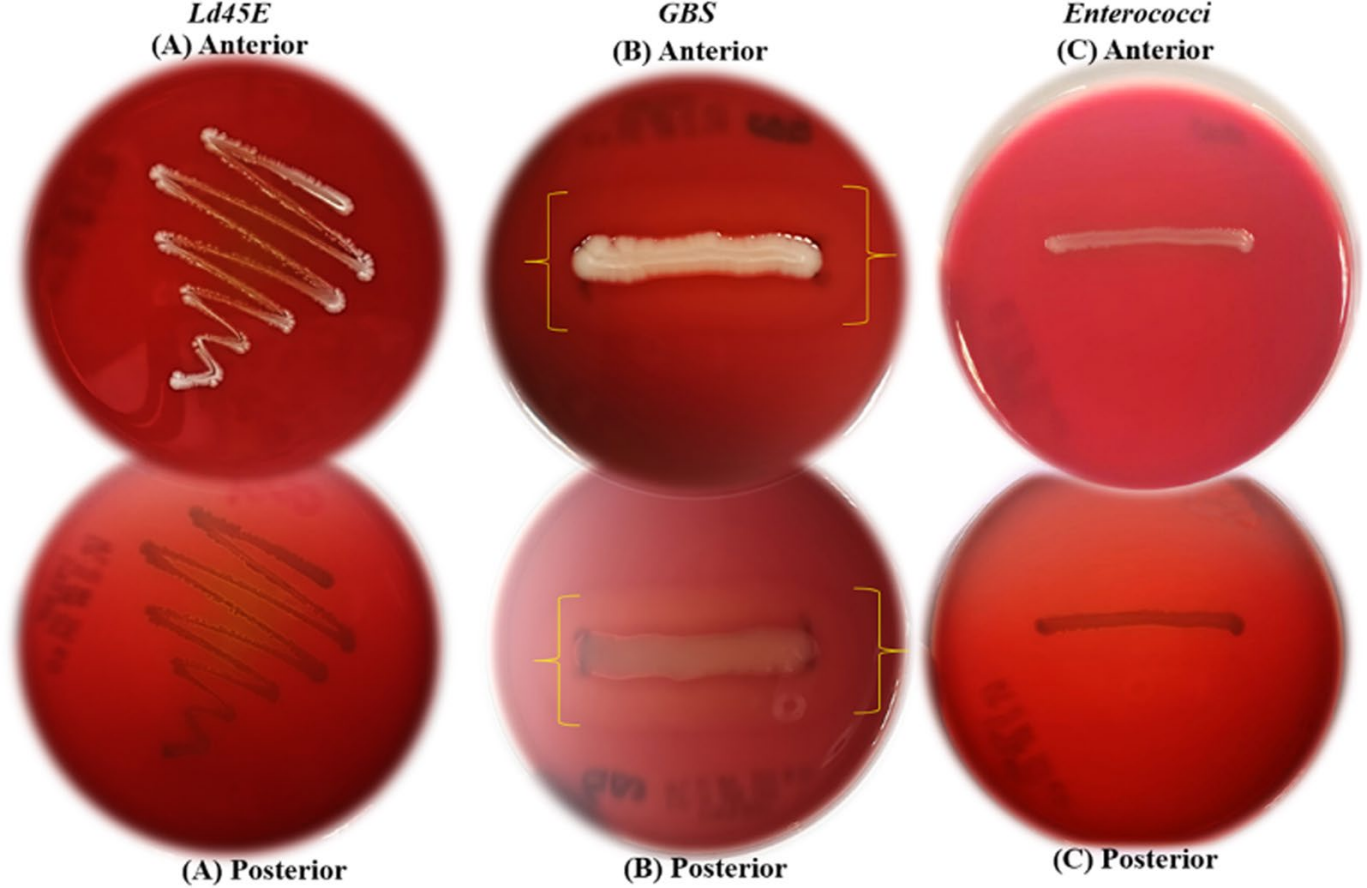
Exhibition of hemolysis activity. **A** the anterior and posterior appearance of *Ld45E* shows no hemolysis activity (γ-hemolysis) on blood agar. **B** the anterior and posterior appearance of Group B *Streptococcus*, the yellow brackets show β-hemolysis activity on blood agar (positive control). **C** the anterior and posterior appearance of *Enterococcus* spp*.* shows no hemolysis activity (γ-hemolysis) on blood agar (negative control)

The *Ld45E* assessment results showed a safety feature of the strain and revealed no positive hemolysis on blood agar. *Ld45E* was a γ-hemolytic strain and did not cause blood lysis. GBS was used as a positive control (β-hemolytic), and *Enterococcus* spp. showed no hemolysis activity (γ-hemolysis) on blood agar (negative control), as shown in (Fig. 4).

### Antibiotic-susceptibility tests

The result of the antibiotic-susceptibility test of *Ld45E* to various antibiotics are shown in Table 2. The inhibition zone was interpreted based on the breakpoints proposed by Charteris et al. [26] and CLSI (2019) guidelines. *Ld45E* was compared with the inhibition-zone reference [26]. The strain exhibited different inhibition zones according to the antibiotic group. (*R*) indicated resistance susceptibility, whereas (*S*) showed sensitive susceptibility to the antibiotics. *Ld45E* with (*I*) had intermediate sensitivity to the antibiotics. Indication of antibiotic-susceptibility test was performed on MRS agar to ensure *Ld45E* proper growth. *Ld45E* was sensitive to the β-lactam-group antibiotics ceftriaxone (30 µg) and cefuroxime (30 µg), the antibiotic of protein inhibitor–mRNA synthesis group doxycycline (30 µg), and the non-β-Lactam ciprofloxacin (30 µg). However, intermediate sensitivity was noticed to levofloxacin (30 µg).

**Table 2:** Antibiotic susceptibility test of *L. delbrueckii* 45E with disk-diffusion method

Classification	Sensitivity measurement as a diameter of inhibition (mm)			
Antibiotic categories	Types of antibiotics	*Ld45E*	*LRC14*	GBS
β-Lactam group	Ceftriaxone (30 µg)	S* (20)	S* (23)	S* (20)
	Cefuroxime (30 µg)	S* (23)	R (8)	S* (20)
	Ampicillin (30 µg)	R (10)	I (16)	S* (22)
Protein inhibitor–mRNA synthesis	Gentamicin (10 µg)	R (13)	I (18)	I (18)
	Kanamycin (10 µg)	R (13)	R (10)	R (10)
	Erythromycin (30 µg)	R (13)	R (4)	R (3)
	Doxycycline (30 µg)	S* (32)	S* (30)	R (10)
Non-β-lactam nucleic acid synthesis inhibitors	Ciprofloxacin (30 µg)	S* (20)	S* (22)	S* (30)
	Trimethoprim-sulfamethoxazole (30 µg)	R (11)	R (9)	R (11)
	Levofloxacin (30 µg)	I (18)	I (15)	R (0)

*LRC14*  *L. reuteri* RC-14, *GBS*  Group B *Streptococcus*

*Inhibition zone of the bacterial-susceptibility test was compared with the inhibition zone provided by Charteris et al. [26]

(S) Sensitive

(R) Resistance

(I) Intermediate sensitivity

#### Autoaggregation capacity

Table 3 shows the percentage of autoaggregation of *Ld45E*. Autoaggregation is generally mediated by the self-recognizing surface structures of the bacteria, such as proteins and exopolysaccharides. Autoaggregation was the first step for biofilm formation, which provides survival sites for LB and pathogenic bacteria. This feature allows the aggregated bacteria to protect themselves from environmental stress and withstand the aggregation, proliferation and colonization of pathogens on the mucus membrane of the host. *Ld45E* in our study showed better autoaggregation affinity than the control group *L. reuteri* RC-14. In a short time, (80 ± 4.02) of *Ld45E* formed precipitate agglutinations under a light microscope expressed as (+ + good aggregation) with a clear solution. By comparison, the control group *L. reuteri* RC-14 exhibited (60 ± 5.07) mixed autoaggregation (± aggregation) with constant turbidity and formed precipitate agglutinations under the light microscope.

**Table 3:** Percentage of autoaggregation exhibited by *Ld45E* after 4 h of incubation

Species	Percentage of autoaggregation at 4 h of incubation
*L. delbrueckii* 45E	80 ± 4.02 (+ +)
*L. reuteri* RC-14 (control)	60 ± 5.07 ( ±)

Values were presented by means of three independent assays ± SD

The score observed under light microscopy was (+ +) good aggregation and ( ±) partial aggregation

### Antimicrobial activity of *Ld45E*

#### Antagonistic effect of *Ld45E* culture using agar plug diffusion method

As shown in Table 4, *Ld45E* exhibited the highest mean of growth-inhibition effect (GIE) against GBS clinical isolate (50.2 ± 1.9 mm) and GBS ATCC 8017 (50.1 ± 2.0*) within 48 h. Incubation of *Ld45E* with the clinical isolate *E. coli* and *E. coli* ATCC 25,922 had GIE a mean equivalent to (30.2 ± 0.4 mm), followed by the clinical isolate of *Klebsiella* spp. and *Klebsiella* spp. ATCC3566 (25 ± 0.2 mm, 24.1 ± 0.2 mm). The minimum inhibitory effects were observed among the clinical isolate *C. parapsilosis* and *C. parapsilosis* ATCC 3434 (19 ± 0.1 and 18.3 ± 0.1 mm). Statistical analysis of the results showed that the inhibitory effects demonstrated by *Ld45E* were significant (*P* < 0.001) and the control group *L. reuteri* RC-14 *P* < 0.01 when both strains encountered GBS.

**Table 4:** Mean diameter of inhibition zones (mm) of *Ld45E* against selected test pathogens

Species	Group B *Streptococcus* clinical isolate	Group B *Streptococcus* ATCC 8017	*Escherichia* *coli* clinical isolate	*Escherichia* *coli* ATCC 25,922	*Klebsiella* spp. clinical isolate	*Klebsiella* spp. ATCC 3566	*Candida parapsilosis* clinical isolate	*Candida parapsilosis* ATCC 3434
*L. delbrueckii* 45E	50.2 ± 1.9*	50.1 ± 2.0*	30.2 ± 0.4	30.2 ± 0.4	25 ± 0.2	24.1 ± 0.2	19 ± 0.1	18.3 ± 0.1
P value	0.001	0.001	0.12	0.12	0.08	0.09	0.56	0.59
*L. reuteri* RC-14 control	22.4 ± 0.9*	23.1 ± 1.2*	18.5 ± 0.6	19 ± 1.8	17 ± 0.8	17.3 ± 0.09	11 ± 0.06	13 ± 0.07
P value	0.01	0.01	0.09	0.09	0.49	0.45	0.19	0.19

Data are presented as the mean ± SD. The finding was based on the average values of three independent assays

*Indicates that the value was significant, p < 0.05 compared with the control, *L. reuteri* RC-14

#### Antagonistic effect of *Ld45E* CFS and CBS using well-diffusion methods

Table 5 indicates the diameter of the inhibition zone on different pathogens at different temperatures by using CFS and CBS. CFS results showed the highest inhibitory effect of *Ld45E* for GBS (42.1 ± 0.2). CFS was active at the temperature of 40 °C and showed the highest GIE at 48 h of incubation among the three CFS (37 °C, 40 °C, 50 °C). These inhibition effects could indicate that the organic material and proteins in the CFS were activated and stable at 40 °C. Moreover, the antimicrobial activity of the bacteriocin produced by *Ld45E* showed a stable inhibitory effect at 37 °C and 40 °C. The highest GIE mean was indicated for GBS (35.3 mm), followed by *E. coli* (33.5 mm), *Klebsiella* spp*.* (32 mm), and *C. parapsilosis* (26.4 mm).

**Table 5:** Inhibitory effect of cell-free supernatant solution and crude bacteriocin of *L. delbrueckii* 45E at different temperatures on selected pathogens

Pathogens	Solution temperatures	Diameter of zone of inhibition (mm)	
		Cell-free supernatant	Crude bacteriocin solution
Group B* Streptococcus*	37 °C	39.6 ± 1.1	35.2 ± 0.5
	40 °C	42.1 ± 0.2	35.3 ± 0.5
	50 °C	35.3 ± 0.8	31.5 ± 0.2
*Escherichia coli*	37 °C	35.1 ± 0.8	32.4 ± 0.3
	40 °C	37.6 ± 0.9	33.5 ± 0.3
	50 °C	30.8 ± 0.7	30 ± 0.3
*Klebsiella* spp*.*	37 °C	30.8 ± 0.7	32 ± 0.3
	40 °C	30.3 ± 0.7	32 ± 0.3
	50 °C	28.5 ± 2.0	30.2 ± 0.3
*Candida parapsilosis*	37 °C	30.5 ± 2.0	25.7 ± 0.6
	40 °C	30.1 ± 0.7	26.4 ± 0.6
	50 °C	29.3 ± 0.7	20.8 ± 0.5

Data are presented as the mean ± SD. The finding was based on the average values of three independent assays

### Co-aggregation and competition assays

#### Co-aggregation ability of *Ld45E* with genitourinary tract pathogens

Table 6 shows the percentage of *Ld45E* co-aggregation against several pathogens. The affinity of *Ld45E* to aggregate with a similar LB strain *L. reuteri* RC-14 (control) expressed the highest co-aggregation percentage (87.5 ± 5.2). *Ld45E* was also co-aggregated firmly with Gram positive GBS (81.2 ± 9.6). However, co-aggregation with Gram negative *E. coli*, *Klebsiella* spp. and the yeast *C. parapsilosis* was relatively low (42.2 ± 20.6, 38.4 ± 24.4, and 12.6 ± 1.3, respectively). Statistical analysis of three readings indicated a significant co-aggregation (p < 0.05) with GBS compared with the control group *L. reuteri* RC-14. The co-aggregation capacity of *Ld45E* could be associated with cell-surface proteins.

**Table 6:** Percentage of *Ld45E* co-aggregation against several pathogens after 4 h of incubation

Species	*Lactobacillus reuteri* RC-14	Group B *Streptococcus*	*Escherichia coli*	*Klebsiella *spp*.*	*Candida parapsilosis*
*Lactobacillus delbrueckii* 45E	87.5 ± 5.2*	81.2 ± 9.6 *	42.2 ± 20.6	38.4 ± 24.4	12.6 ± 1.3
P value	0.01	0.05	0.09	0.11	0.30

Values were the means of three independent assays ± SD; * shows that the value was significant at p < 0.05 compared with the control group

#### Competition ability of *Ld45E* with genitourinary tract pathogens

**Fig. 5:**
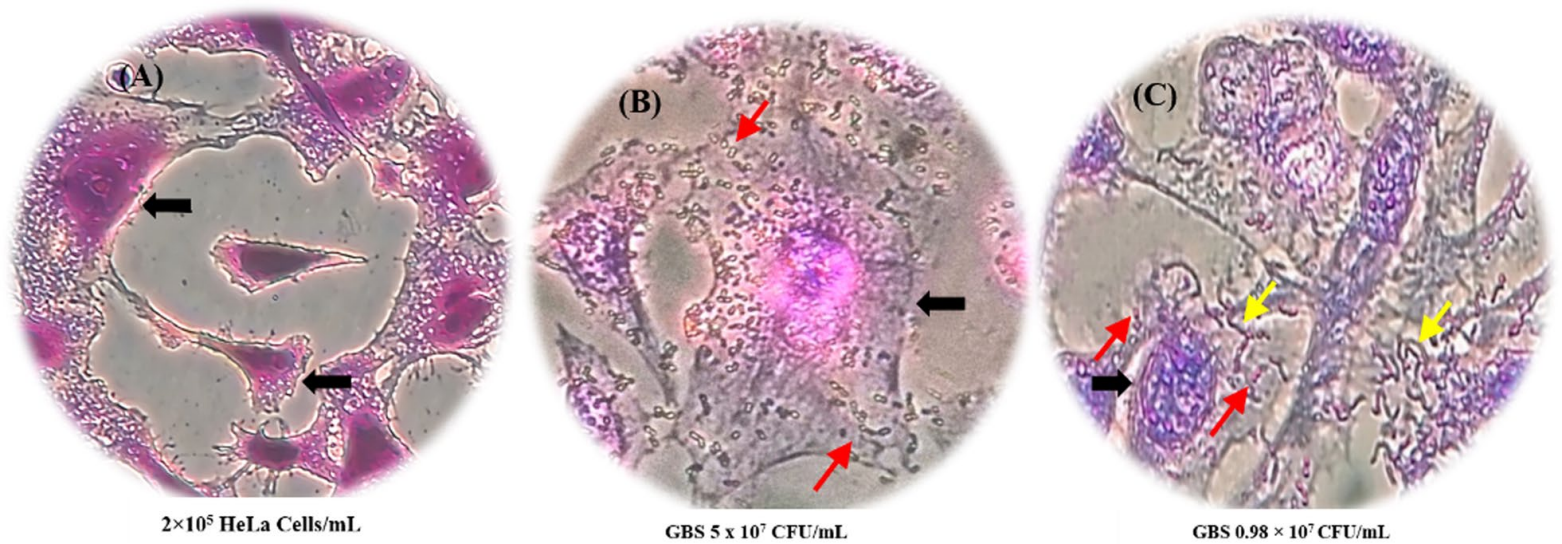
Competition assay of *Ld45E* with GBS on HeLa cells. The black arrows in (**A**) show the (blank) HeLa cells (purple stain) after incubation for 24 h at 37 °C in a 5% CO_2_ incubator. The red arrows in (**B**) illustrate the (control group) GBS (pinkish stain) aggregation on HeLa cells after incubation for 4 h at 37 °C. The yellow arrows in (**C**) indicate the (*Bacilli* strain) *Ld45E* (purple stain) reduces the aggregation of GBS (pinkish stain) indicated by the red arrows, on HeLa cells indicated by the black arrow stained purple with crystal violet. Viewed under inverted microscopy magnification 40 ×

Table 7 indicates the competition ability of *Ld45E* with pathogens on HeLa cells. The competitive property of LB inhibited the pathogenic attachment onto the cell surface, either by occupying the cell surface receptor or interfering with the microorganism’s nutritional source. Our LB strain *Ld45E* and pathogens were incubated simultaneously in a 1:1 ratio of approximately (5 × 10^7^ CFU/mL) for each bacteria. Table 7 summarizes two comparable results. The ability of *Ld45E* to adhere onto HeLa cells in the presence of GBS was 1.5 × 10^7^ CFU/mL of the total incubated *Ld45E* (5 × 10^7^ CFU/mL). Similarly, *Ld45E* adherence onto HeLa cells was relatively low in the presence of *E. coli*, *Klebsiella* spp., and *C. parapsilosis* (1.3 × 10^7^, 1.1 × 10^7^, and 0.4 × 10^7^ CFU/mL), respectively. However, *Ld45E* showed a competitive ability over similar Gram positive GBS, where 80% GBS was eliminated from adhesion onto HeLa cells as shown in Fig. 5. Additionally, the affinity of *Ld45E* to compete with *E. coli* was (76%) and *Klebsiella* spp. (72%). Conversely, *C. parapsilosis* debarred the attachment of *Ld45E* with a relatively low competitive ability equivalent to 58% on HeLa cells.

**Table 7:** Competition of *Ld45E* with several pathogens on HeLa cells

CFU/mL				
Species	Group B* Streptococcus*	*Escherichia coli*	*Klebsiella* spp*.*	*Candida parapsilosis*
*L. delbrueckii* 45E	1.5 × 10^7^	1.3 × 10^7^	1.1 × 10^7^	0.4 × 10^7^
Adherence rate	30%	26%	22%	8%
Competition rate%	80%	76%	72%	58%

### Effect of *Ld45E* on IL-17 secretion

**Fig. 6:**
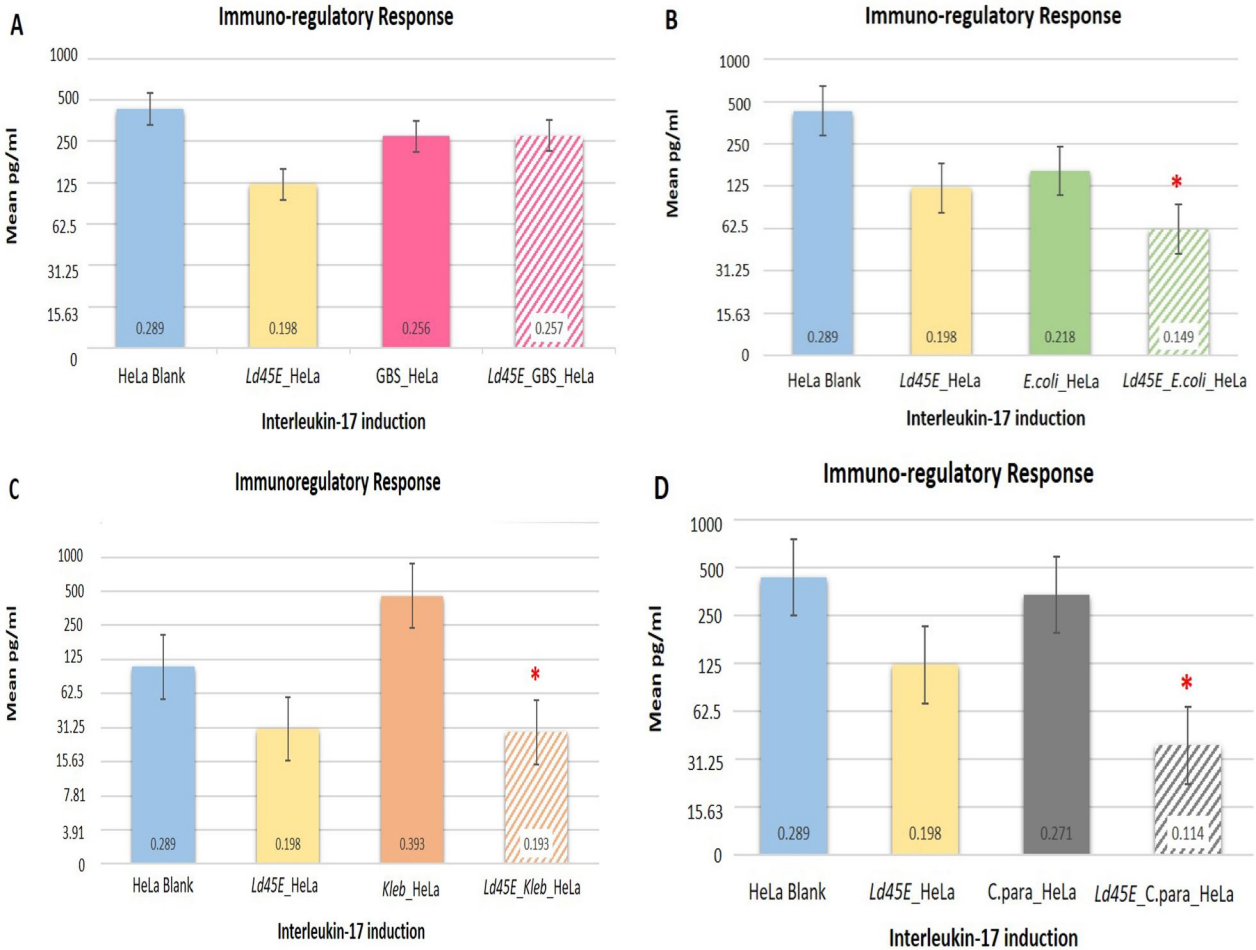
Induction of interleukine-17 with the application of *Ld45E* in the presence of different pathogens (left–right columns). (*) indicates the significant reduction of IL-17 production. Column (1): expresses the supernatant of HeLa cells cultured alone (0.289 pg/mL). Column (2): shows the supernatant of *Ld45E* alone on HeLa cells (0.198 pg/mL). **A** Column (3): shows the supernatant of GBS (control) incubated alone on HeLa (0.256 pg/mL), column (4): shows the supernatant of *Ld45E* incubated with Group B *Streptococcus* (0.257 pg/mL). *Ld45E* does not reduce IL-17 production. **B** Column (3): shows the supernatant of *E. coli* (control) incubated alone on HeLa cells (0.218 pg/mL), column (4): shows the supernatant of *Ld45E* significantly reduces IL-17 production when incubated with *E. coli* on HeLa cells (0.149 pg/mL). **C** Column (3): shows the supernatant of *Klebsiella* spp. incubated alone on HeLa cells (0.393 pg/mL), column (4): shows the supernatant of *Ld45E* significantly reduces IL-17 production when incubated with *Klebsiella* spp. on HeLa cells (0.193 pg/mL). **D** Column (3): shows the supernatant of *C. parapsilosis* incubated alone on HeLa cells (0.271 pg/mL), column (4): shows the supernatant of *Ld45E* significantly reduces IL-17 production when incubated with *C. parapsilosis* on HeLa cells (0.114 pg/mL)

The current study evaluated proinflammatory proteins in response to genitourinary tract pathogens. To better understand the role of *Ld45E* with IL-17 production, pathogens were incubated alone with HeLa cells prior to *Ld45E* application. IL-17 production was measured and compared with the pretreated pathogens with *Ld45E*. HeLa cells were stimulated to produce IL-17 incubated with several pathogens for 24 h. *E. coli* induced (0.218 OD_450_ nm) absorption under a spectrophotometer, equivalent to the concentration of 500 pg/mL, compared with the absorbance of GBS (0.256 pg/mL), *Klebsiella* spp. (0.393 pg/mL), and *C. parapsilosis* (0.271 pg/mL), equivalent to the highest IL-17 concentration of 1000 pg/mL, provided in the protocol. The pre-incubation of HeLa cells with *Ld45E* alone before incubation with pathogens induced a low production of IL-17 (0.198 OD_450_ nm), equivalent to the concentration of 250 pg/mL. In separate cultures, pretreating HeLa cells with *Ld45* significantly suppressed IL-17 production in response to *E. coli* (0.149 OD_450_ nm) equivalent to (62.5 pg/mL), *Klebsiella* spp. (0.193 OD_450_ nm) equivalent to (250 pg/mL), and *C. parapsilosis* (0.114 OD_450_ nm) equivalent to (31.25 pg/mL). No significant reduction in IL-17 production was observed with GBS (0.257 OD_450_ nm) equivalent to (1000 pg/mL), as illustrated in (Fig. 6A). In our study, *Ld45E* cannot regulate IL-17 production when incubated with GBS. Conversely, *Ld45E* downregulated IL-17 production with *E. coli*, *Klebsiella* spp., and *C. parapsilosis*.

## Discussion

### Characteristics of *Ld45E* probiotic profile

#### Morphology of *Ld45E*

The study of cell morphology highlights some points of interest, which premise the mechanism of action of LB strains [17]*.* In this study, *Ld45E* was large bacilli, as shown in (Fig. 1), which reflected the ability of the strain to withstand different pH values and adhere firmly onto HeLa cells. Nwamaioha and Ibrahim explored the possibility of growth media affecting the colonies and size of *L. delbrueckii* spp., and MRS was the optimum medium in providing the essential elements to enhance the species’ growth [34]. Furthermore, the size and morphologies of *Ld45E* changed with the incubation with other pathogens; According to Rajab et al., the large size (long and thick) of the LB strain can justify the slow growth of its colonies, which was noticed among *Ld45E* compared with the control *L. reuteri* RC-14 [17]. Inevitably, the morphology features of *Ld45E* clarified the strong adhesion capacity of the species onto HeLa cells when incubated with the pathogens. The findings of this study agreed with those of Nwamaioha and Ibrahim, who found that the large size of *L. delbrueckii* spp. is significantly correlated with aggregation activity compared with *L. reuteri* and other small-size *Lactobacilli* spp. [34]. Previous studies have indicated that LB are highly sensitive to physical changes such as freezing, UV radiation, drying, X-rays, heat, and storage, which directly influence their morphologies and size. Their characteristics to lose their shape and viability quickly are also affected [31].

#### The pH tolerance of the *Ld45E*

A condition simulating the vaginal medium was achieved to evaluate the stability of *Ld45E* in different pH ranges. The human vaginal fluid was affected by several factors, including interactions of the vaginal medium with topical contraceptives, prophylactic, or therapeutic drugs [21]. Besides, the pH changes during the menstrual period, causing an alteration in vaginal microbiota [35] and leading to the growth of pathogens and increased pH [36]. Accordingly, *Ld45E* was tested at different pH values to assess its ability to withstand different vaginal pH values under the previous conditions. Linhares et al. illustrated that LB could maintain the vagina medium at a constant pH < 4.5. The ability of LB to tolerate different pH ranges nominates them to be a probiotic candidate [2].

In the current study, *Ld45E* grew in a medium simulating the vaginal pH (4.0–4.5). The species tolerated the higher pH (6–7) and showed good growth within 6 h of incubation. *Ld45E* could constantly grow within pH 3–7, which was considered a good sign for LB to withstand the alteration of the vaginal pH in different health conditions during 12 h of incubation. After 24 h of incubation, *Ld45E* at pH 4 and 4.5 could survive compared with other pH ranges, wherein the growth of *Ld45E* was visibly decreased. This reduction could be referred to lack of nutrients in the medium. According to Pan et al., interference of the incubated medium (MRS) could decrease the efficacy of *Ld45E* growth compared with simulated vaginal fluid for a long incubation time [37]. Incubation of *Ld45E* in pH (3 and 3.5) showed decreased growth within the first 6 and 24 h of incubation, and constant growth was observed within 12 h of incubation, compared with the control group *L. reuteri* RC-14, which showed no effects. The interpretation of this could be due to the enormous cell size of *Ld45E* may develop acid shock in the early growth phase until it slowly adapts to the medium [17].

Acid tolerance is essentially related to strain-specific properties, where they can maintain a constant pH gradient between the medium pH and the cytoplasm of the surrounding environment [38]. Nemska et al. indicated that the relatively low percentage of Lactobacilli (LB) survival under acid conditions (pH 2.0, 3.0, and 4.0) is likely attributable to the incubation period, which is eight times longer than a normal passage through the gastrointestinal tract (GIT) in-vivo study [39]. Our methodologies showed a substantial difference in the growth parameters optical density OD and the number of vital cell colony-forming units (CFU) recorded in the investigated strains. This study finding was in contrast to previous research findings because we measured the pH tolerance within multiple ranges [5, 39, 40].

#### Adhesion ability of *Ld45E* onto HeLa cells

The adhesion of LB onto the epithelial cells is one of the most critical parameters that identify the probiotic features of a microorganism [22]. In healthy genitourinary tract, LB species are believed to exhibit a defense mechanism against pathogen colonization either by occupying the bacteria-binding site on the epithelial cells or challenging the nutrition source [41]. During an infection, the environment of the LB depletes, making it impossible for the LB to displace or compete with the pathogens for the same receptors. Thus, LB strains that block the adherence of genitourinary pathogens possess sound antimicrobial effects either by excluding, displacing, or competing with the receptor-binding sites of the adhered pathogens [22, 41]. In the current research, our LB strain *Ld45E* showed strong adhesion properties onto HeLa cells when incubated alone on the HeLa cell line. This finding agreed with that of Carmo et al., who demonstrated the ability of *Lactobacillus* spp. to adhere onto the vaginal epithelial cells [22], which in turn form a biological barrier against the colonization of the pathogenic bacteria [42]. The adherence ability of LB onto epithelial cells reportedly refers to the presence of multiple components in the bacterial cell surface, such as glycoproteins and carbohydrates [43]. Large LB species also display better adherence onto the cell line than small-size species [17]. Adhesion onto the cellular surface is also multifactorial, involving different interaction mechanisms between LB and cell-surface components. For instance, specific mechanisms occur in the surface molecules of the outer membrane proteins such as exopolysaccharides (EPPs) [44], lipoteichoic acids, peptidoglycans [45], glycosaminoglycans that can lead to species-specific adhesion [46]. Conversely, nonspecific mechanisms are displayed by electrostatic and hydrophobic interactions [22]. Hence, the ideal LB for probiotic nomination were those exhibiting a high binding affinity to the vaginal mucus membrane that facilitates their permanent growth in the female genital tract.

#### Hemolytic activity

The long history of using LB strains with rare side effects made them safe for use. Seldomly clinical conditions such as bacteraemia and endocarditis can be caused by some strains of LB, which can directly refer to the patient’s health status; for instant, vancomycin-resistant *L. rhamnosus* was reported in a 14-year-old girl with leukemia [47]. Therefore, the hemolytic activity and antibiotics resistance test were performed for the safety assessment of *Ld45E*. The prerequisite test indicated that the tested LB did not cause lysis to the erythrocytes (RBCs) on the incubated blood media. Conversely, complete lysis (β-hemolysis) was registered in the positive controls GBS. According to a previous study, most of the isolated LB from the human vagina showed no hemolytic activity [23]. In the present study, the hemolytic activity of *Ld45E* showed slight discoloration on the blood agar, indicating the strain had γ- hemolytic activity and considered safe. This result supported the finding of Estifanos that *L. delbrueckii* spp. are safe as they do not exhibit (α or β) hemolytic activity [5]. Conversely, Casarotti et al. reported that *L. delbrueckii* subsp. *bulgaricus* SJRP50, SJRP76, and SJRP149 and *L. casei* SJRP141. show partial hemolysis, meaning hemolytic activity was a strain-specific trait [48].

#### Antibiotic susceptibility

The antibiotic-susceptibility test was used to ensure the beneficial effects of probiotics, which could be used as a medical treatment that, in turn, may exhibit side effects on their hosts. The antibiotics were selected according to the most prescribed treatment for vaginitis. In other words, these medications can be safely used if an overgrowth of the probiotic candidate occurs during the treatment of vaginal pathogens. Based on the current results as presented in Table 4, *Ld45E* was resistant to five antibiotics of different inhibitory mechanisms, including ampicillin (30 µg), gentamicin (10 µg), kanamycin (10 µg), erythromycin (30 µg), and trimethoprim-sulfamethoxazole STD (30 µg). Previous studies have suggested that susceptibility and resistance to different antibiotics may differ among LB strains. LB become resistant to some antibiotics such as kanamycin, amikacin, rifampicin, ciprofloxacin, and vancomycin owing to intrinsic resistance of the strain [49]. Moreover, resistance to chloramphenicol, erythromycin, clindamycin, and tetracycline was due to the presence of transferable resistance genes [50].

#### Autoaggregation capacity of *Ld45E*

Autoaggregation of *L. delbrueckii* and *LB reuteri* was previously studied for their cell-surface proteins that promote aggregation effects [51]. Our result showed a significant autoaggregation (+ +) property of *Ld45E* compared with the control group *L. reuteri* RC-14. This finding was in agreement with Collado et al., who indicated that most lactic acid bacteria (LAB) have high adhesion properties and expressed good autoaggregation between the same strains [52]. Moreover, a study by Choo et al. compiled that autoaggregation was not only strain-dependent but also time-dependent [12].

### Antimicrobial Activity of *Ld45E*

#### Antagonistic effect of *Ld45E* culture using agar plug diffusion method

The results of the current study illustrated the inhibitory effects of *Ld45E* against tested pathogens. The highest inhibitory effect was identified among similar Gram positive GBS*,* followed by Gram negative *E. coli*, *Klebsiella* spp. and the yeast *C. parapsilosis.* The control *L. reuteri* RC-14 showed a lower inhibition effect than *Ld45E.* The GIE may vary depending on the bacterial strains, species-dependence and strain origin [53]. Based on Abedi et al. results, *L. delbrueckii* spp. showed highly inhibitory effects against *E. coli* by secreting metabolites that reduce the pH, making the surrounding environment unfavorable for pathogenic colonization [54]. Conversely, a study by Lopes et al. stated that *L. delbrueckii* spp. exhibited no antimicrobial effect on pathogens because the strain lacked organic acid production [55]. The findings of Li et al. indicated the antifungal activity of *L. crispatus* and *L. delbrueckii* against vaginal candidiasis [8]. Both *Lactobacilli* spp. were able to compete for colonisation of the vaginal epithelial cells and control the overgrowth of yeast on the vaginal epithelial cells [8]. Another study by Santos et al. demonstrated that LB generally are origin-dependent organisms [14].

#### Antagonistic effect of *Ld45E* CFS and CBS using well-diffusion method

CFS and CBSs were not identified for their active components; however, the solutions were tested for their thermo-sensitivity at three different temperatures (30 °C, 40 °C and 50 °C) to determine the temperature of heat-stable activity. Heat-labile substances produced by LB could be resistant to proteinase K cleavage, affecting LB’s biofilm matrix [56].

The diffused CFS solution exhibited a variable degree of antimicrobial activity on various tested pathogens on well-diffusion agar. CFS of *Ld45E* showed a good inhibitory effect among GBS, whereas Gram negative bacteria (*E. coli* and *Klebsiella* spp.) and *C. parapsilosis* produced the lowest inhibitory rate. Previous researchers have found the high activity of probiotic lactic acid bacteria (LAB) supernatant on pathogenic bacteria and attribute them usually to the production of organic compounds [16]. According to Lim et al., CFS contains more than 19 substances known for their antimicrobial activities as high-intensity spot proteins [57]. Our results agreed with Muhsin et al. findings that related the activity of the LB supernatant to the classification of Gram bacteria, which showed the highest antimicrobial effect on similar Gram stain bacteria [16].

On the other hand, LB produce bacteriocin within 24–30 h of their exponential phase and reach its maximum level during the stationary phase of the strain growth curve [58]. Bacteriocin production highly depends on the growth-medium composition [15]. According to the current results, the bacteriocin from *Ld45E* was extracted within 30 h of incubation and exposed to different temperature ranges to minimize the antimicrobial activity of enzymes such as protease [59]. Bacteriocin exhibited similar growth of inhibition effect (35.2–30 mm) in different temperature ranges (37–50 °C) among Gram positive GBS and Gram negative *E. coli, Klebsiella* spp., but a low GIE on *C. parapsilosis* (26.4 mm). These could be referred to the CB mechanism in similar Gram positive [60] or owing to the antibacterial peptides and proteins that were released extracellularly [56]. Our findings paralleled those of the literature [58], where CB affected Gram positive bacteria and displayed a low effect on the Gram negative bacteria except for *Pseudomonas aeruginosa* [16]*.* Our findings manifested the properties of *Ld45E* in the medical field, where stable effects of bacteriocins at 40 °C indicated its ability to withstand the feverish condition of the patients. Storage of CB at 4 °C remained stable and showed similar GIE for a few days. Stability at low temperatures may be the most suitable preservation and storage technique in the future. Consistently, bacteriocin stability at high temperatures is a significant factor that determines its potential use as a food preservative.

### Co-aggregation and competition assays

#### Co-aggregation ability of *Ld45E* with genitourinary tract pathogens

Co-aggregation of genetically different bacterial strains was critical for several ecological niches, and only some *Lactobacilli* spp. possess this feature. Thus, non-aggregating *Lactobacilli* spp. cannot induce self-or co-aggregation even with the presence of aggregation factors, such as peptide on the host surface and bacteria or carbohydrate receptors on the yeast [22].

LB expressed different co-aggregation activities based on several factors. Previous studies indicated that co-aggregation depends on the origin of the tested LB and the source of pathogenic strains [61, 62]. The present research disagreed with the findings of Kowalska et al. and Zawistowska-Rojek et al. [63, 64], whose clinical studies on the LB *L. casei* LOCK 1132 show low co-aggregation ability (10%) compared with ATCC *LB rhamnosus* LOCK 1131 and exhibit relatively higher co-aggregation rate (80%) to ATCC pathogen *S. typhimurium* ATCC 13,311. This finding could be due to a strain-specific adhesion, which was in accordance with previous studies by Pino et al. [19] and Younes et al. [62]. Other factors that promoted co-aggregation include the time of incubation with the pathogens, as well as LB’s length, where longer LB have higher co-aggregation ability than those with short length [17, 64].

#### Competition ability of *Ld45E* with genitourinary tract pathogens

*L. delbrueckii* 45E was able to reduce around (80%) of GBS, *E. coli* (76%) and *Klebsiella* spp. (72%) at the same time, approximately 30%, 26%, and 22% of *Ld45E* were able to adhere onto HeLa cells, respectively, with each pathogen. Conversely, only 8% of *Ld45E* were able to attach to HeLa cells in the presence of *C. parapsilosis* and eliminate 58% of them only. Our study was in agreement with Abedi et al. [54], *L. delbrueckii* spp. were able to reduce more than 50% of *E. coli,* and 28.6% were able to adhere onto Caco cells. The possible mechanisms of competing with these species could be competition for common adhesion receptors on the host cells, as revealed by Meng et al. [65], or the excretion of substances that suppress the adherence of pathogens, as described by Barzegari et al. [66]. Another study has shown that LB species were able to significantly inhibit the colonization and biofilm formation of Gram positive *Staphylococcus aureus* and uropathogenic *E. coli* on a rubber tube, indicating that probiotics can prevent bacterial adhesion onto uroepithelial cells and displace adherent uropathogens from urinary devices materials [67]. From another perspective, nutritional balance in human organs is considered a second type of immunity trigger, known as nutritional immunity [68]. Some LB species express a specific gene, e.g., “mntH gene,” that displays a distinct phylogenetic pattern within a *LB* genus and causes nutritional depletion in the respective parts of the human body. Iron, zinc, and manganese are some nutritional minerals that affect the growth of yeast and bacteria in the human body. According to Siedler et al., LB can inhibit the growth of pathogenic fungi expressing the gene depletion that reduces the amount of iron and manganese in the host [69].

### Effect of *Ld45E* on IL-17 secretion

T helper cells are the main producer of IL-17 in addition to other T cells subsets, such as γδT and natural killer T. IL-17 can modulate the immunological function against infections by inducing the proinflammatory cytokine, anti-pathogenic peptide, and chemokine secretion by the responder cells or enhance the disease severity, playing a protective role in genital innate immunity to intracellular pathogens such as bacteria, fungi, and viruses [9]. The concentrations of genital IL-17 or 17A are closely associated with other inflammatory cytokines and growth factors. The T-helper type 17 cells produce this cytokine in response to the proinflammatory with the stimulation of IL-23. Bacterial vaginosis, primarily *chlamydia* and *N. gonorrhea*, induce increased IL-17 concentrations compared with the healthy vagina. A previous study has indicated decreased IL-17 concentration among women with candidiasis, whereas IL-17 concentration shows no significant association with a genital viral infection such as herpes simplex virus II and HIV [70].

Our recent research explored the antimicrobial activity and the safety properties of *Ld45E*. It also focused on understanding the beneficial effects of the strain as a potential probiotic candidate to regulate the immune-related functions of host cells, by measuring the induced IL-17 that represents the tolerogenic response of T helper cells. In this study, incubation of HeLa cells with the pathogens alone induced proinflammatory protein (IL-17) production. *Ld45E* incubation showed significant downregulation of IL-17 in response to the presence of pathogens, including *E. coli*, *Klebsiella* spp. and *C. parapsilosis* and no difference in IL-17 production was marked for GBS.

Earlier studies have addressed the immunoregulatory ability of *L. delbrueckii* spp. *bulgaricus* strain to reduce the production of different inflammatory proteins such as IL-10, IL-4, IFNγ, and TNF-α in an animal model [71]. Other studies have demonstrated the immunomodulation properties of *L. delbrueckii* species, which could be related to the numerous characteristics of a rapidly evolving genome. The most efficient strains can lower NF-_k_B activity to a close to the background level recorded in the absence of TNF-a or bacteria. Almost all tested *L. delbrueckii* strains exhibited a stronger anti-inflammatory impact than the commensal strains *L. gasseri* ATCC 33,323, *L. acidophilus* NCFM, and L. *salivariu*s 33 [72–74].

Our findings were similar to previous ones suggesting that *L. delbrueckii* spp. could regulate the release of inflammatory cytokines [8]. This observation agreed with previous ones on probiotic strains that counteract the molecular events leading to T-cell activation [75].

Feasibly the effect of *Ld45E* on IL-17 induction depended on the exposed proteins to the transcription factor. Santos Rocha et al. studied 20 strains of *L. delbrueckii* spp. for their strain-dependent anti-inflammatory features [72]. The result proved that the immunomodulation effects of *L. delbrueckii* spp. *bulgaricus* depended on the exposed-proteins of the bacterial surface that influence the center of the NF-_*k*_B activation pathway. *L. delbrueckii* spp. *bulgaricus* herein were compared with the other tested probiotic strains *L. salivarius*, *L. acidophilus*, and *L. gasseri* to assess their potential anti-inflammatory traits. However, the mechanism of action of IL-17 was suggested for future study.

*Ld45E* were unable to downregulate IL-17 production in the presence of GBS. The reason could be the microbe-associated molecular patterns (MAMPs) in bacteria, which were recognized by the host cell’s pattern-recognition receptors (PRRs). Host cells that express a specific class of PRRs known as toll‑like receptors (TLRs) can activate the mechanism of microbe-specific innate and adaptive immune responses [76]. This interaction was responsible for distinguishing between commensal and pathogenic bacteria, where Gram positive bacteria usually share similar (TLRs). Shibata et al. found that high production of IL-17A in response to bacterial infection was related to early infection of *E. coli* [77]*. C. albicans* has also been found to trigger inflammatory reactions in in vitro and in vivomodels, and several LB strains downregulated cytokine levels [75]. Furthermore, during *Klebsiella pneumonia* infection, cytokine-like IL-23 triggered the induction of IL-17 [78]. According to Yan and Polk, LB-derived proteins and acidification of lactic acid could be suppressive agents that modulate the immune cells’ signaling pathway and mediate innate immunity by inhibiting cytokine production [79, 80].

### Strength and limitation

To our knowledge, our study was the first to investigate the immunoregulation ability of a particular *LB* in the presence of different genital pathogenic bacteria, such as GBS, *E. coli*, *Klebsiella* spp., and *C. parapsilosis*. However, the immunomodulatory mechanisms of LB are more complicated and multifactorial depending on strain-specific and host-associated factors such as estrogen status. Certainly, the pathogen’s invasion induces inflammatory responses, which are triggered when a damaged tissue releases molecules that activate pattern-recognition receptors. For instance, *Gardnerella vaginalis* toxin (vaginolysin) is reportedly cytolytic to host cells, which activates the p38 mitogen-activated-protein kinase pathway that elevates IL-8 production [81].

This study also had some limitations. Based on the current result, the LB strain showed different outcomes based on time of incubation, temperature and pH changes. The investigated properties of *Ld45E* could be sufficient for an in vitro study, but for the selection as a probiotic candidate, an animal model should be performed for different time interval incubation, temperature, and pH changes as the species exhibited different characteristics accordingly. The antimicrobial components of the isolated CFS and CB need to be analyzed to reveal the enzymatic mechanism of action. This analysis will illustrate the nature of *Ld45E* to challenge different pathogens based on their proteolytic and non-proteolytic enzymes.

More details are required for the antibiotic-susceptibility test, which can help prescribe the probiotic candidate with the resistance drug of choice to avoid destroying the vaginal ecosystem. Studies on resistance genes and genes encoding adhesion and colonization factors are desirable for LB adherence onto the cells’ layers.

## Conclusions

Vaginal probiotics could be a recommended alternative therapy to antibiotics owing to their antimicrobial effects and strong elimination properties of the pathogens’ attachment onto the epithelial cells. Therefore, LB that can colonize the vaginal epithelium are crucial to preventing the invasion of pathogens and maintaining healthy genital ecological balance. This study focused more on the nature of the species and its isolated solutions rather than the compounds. Another interesting feature of *Ld45E* is its ability to withstand pH similar to the vaginal acid range of 4.5–5.5. This feature could be due to the strain’s natural presence in a woman’s vagina. Infection with pathogens such as GBS may not stimulate inflammation in the host cells, which could induce ultrastructural changes in cells and inhibit the desmosome-specialized structure of the cell membrane that facilitates cell–cell adhesion. This procedure may eventually reduce the adhesive strength of the cell–cell and the bacteria–cell over time. To summarize, *Ld45E* exerted strong antimicrobial effects against genital pathogens and induced good immunoregulation, thereby supporting our hypothesis of the strain’s potential as a probiotic candidate for adjunct therapy for vaginal infection.

The outcomes of this research are relevant to adjunct therapeutic development, highlighting the value of LB isolates of the exact microbiota origin to evaluate different aspects of probiotic selection. Hence, studying the characteristics and the inflammatory properties provided a better understanding of the potential vaginal LB. However, further investigation is required on the mechanism of action such as LB’s role in the immuno-modulatory response to the triggering mechanism, as well as the inverse and reverse association of the cell-wall peptidoglycan, cell-wall proteins, and cell-membrane receptors. In this regard, molecular studies are indeed crucial.

## Data Availability

The authors confirm that the data supporting the findings of this study are available within the article.
